# Clinical Characteristics of Seizures and Course of Epilepsy in Children with Neurofibromatosis Type 1—A Tertiary Center Experience in a Cohort of 118 Children †

**DOI:** 10.3390/diagnostics15212690

**Published:** 2025-10-24

**Authors:** Ružica Kravljanac, Jovana Beđik, Irene Bruno, Biljana Vučetić Tadić, Sofija Popović, Vladimir Oparnica, Pavle Kravljanac

**Affiliations:** 1 Institute for Mother and Child Healthcare of Serbia, 11070 Belgrade, Serbia; jovanabedjik@gmail.com (J.B.); biljanavucetic74@gmail.com (B.V.T.); sofiapopovic20@gmail.com (S.P.); vladaopa@gmail.com (V.O.); 2 Faculty of Medicine, University of Belgrade, 11000 Belgrade, Serbia; 3 Institute for Maternal and Child Health, IRCCS, Burlo Garofolo, 34137 Trieste, Italy; irene.bruno@burlo.trieste.it; 4 Chemistry Research Laboratory, Department of Chemistry, University of Oxford, Oxford OX3 9DU, UK; pkravljanac@gmail.com

**Keywords:** NF1, epilepsy, seizures, status epilepticus, children

## Abstract

**Background/Objectives**: This study aimed to improve the knowledge of seizures and epilepsy in children with neurofibromatosis type 1 (NF1) by evaluating the frequency, clinical characteristics, and risk for epilepsy in children with NF1. **Methods**: The retrospective study included all NF1 patients aged one month to 18 years treated at the Institute from 2010 to 2025, who experienced seizures. Diagnosis of NF1 was made using revised diagnostic criteria from 2021. The seizures were classified according to the ILAE classification, updated in 2025. Further parameters were analyzed: sex, age at seizure onset, type and duration, electroencephalographic (EEG) findings, brain magnetic-resonance imaging (MRI), anti-seizure medications (ASMs), treatment response, and outcome. The patients were divided into two groups: epilepsy—NF1 patients (14) and non-epilepsy—NF1 patients (104). To evaluate the predictive factors for epilepsy in NF1 patients, univariate and multivariate logistic regression analyses were performed. **Results**: The study included 118 children with NF1. In 14 children (11.9%), a diagnosis of epilepsy was established. Ten of 14 children (71.4%) experienced SE. Good seizure control was achieved in 13/14. We found statistically significant predictive values of hydrocephalus (*p* = 0.001), brain atrophy (*p* = 0.002), and vasculopathy (*p* = 0.015) for epilepsy in children with NF1. **Conclusions**: In our cohort, the frequency of epilepsy in NF children was at least ten times higher than in the general population. The predictive factors for epilepsy were hydrocephalus, brain atrophy, and vasculopathy. Recommendations for rescue medication and parental education are important, since status epilepticus occurs in a high percentage of NF1 patients with epilepsy.

## 1. Introduction

Neurofibromatosis type 1 (NF1) is a multisystem genetic disorder caused by mutations in a tumor suppressor gene located on chromosome 17 (17q11.2) leading to altered function of the neurofibromin protein [1]. This is one of the most common autosomal dominant disorders, with an estimated minimum prevalence between 1 and 3000 and 1 in 4000 people and an incidence of 1 in 2500 births [2,3]. The clinical criteria for diagnosis include skin hyperpigmentation, axillary and inguinal freckles, Lisch nodules or choroidal abnormalities, optic glioma, neurofibromas, and distinctive osseous lesion [4]. Numerous neurological disorders are part of the clinical presentation in NF1 patients, although they are not included in the diagnostic criteria: macrocephaly, migraine-like headache, intellectual disability, attention deficit hyperactivity disorder (ADHD), cognitive and motor impairment, epilepsy, brain tumors, intracranial malformations, aneurysms, and moyamoya syndrome [5].

The prevalence of epilepsy in patients with NF1 is at least ten times higher than that of the general population with varying rates depending on the age at onset, ranging from 4% to 14% [6]. Data from the pediatric literature suggest that up to 17% of patients with NF1 present with seizures, and about 20% will have one epileptic seizure during their lifetime [7,8,9]. Seizure control in patients with NF1 is variable. About 90% of patients are treated with at least one anti-seizure medication, while 36% require treatment with two or more ASMs [10]. There is no evidence on the mechanisms of epileptogenesis in neurofibromatosis. In the majority of patients, epilepsy is associated with intracranial lesions, but 15–50% do not correlate with brain abnormalities [6,10,11].

The aim of our study was to improve the knowledge of seizures and epilepsy in children with NF1 by evaluating the frequency, clinical characteristics of seizures, course, and biomarkers for epilepsy in children with NF1. The purpose of this study is to evaluate clinical characteristics of seizures, course, and biomarkers for epilepsy in children with neurofibromatosis type 1 (NF1).

## 2. Materials and Methods

The retrospective study included all patients with NF1 aged one month to 18 years treated at the Institute from June 2010 to June 2025, who experienced at least one seizure. Informed consent was obtained from all identifiable human participants. Diagnosis of NF1 was made using the revised diagnostic criteria from 2021 [4]. Diagnosis of epilepsy was established in children with at least two unprovoked seizures [12]. A new definition and classification (convulsive vs. non convulsive) of status epilepticus (SE) was used (Trinka et al., 2015) [13]. The seizures were classified according to the International League Against Epilepsy (ILAE) classification system [14], updated in 2025 [15] into four main classes: focal, generalized, unknown (whether focal or generalized), and unclassified. Antiseizure medication was recommended in the cases with recurrent unprovoked seizures and an established diagnosis of epilepsy. Further parameters were analyzed: sex, age at the time of onset of the first seizure, type and duration of the seizures, provocation, used rescue and ASMs, response to the treatment and outcome (good or poor epilepsy control), and associated brain MRI abnormalities. Good seizure control is defined when the seizure-free period lasted for at least 12 months of follow-up. Standard video EEG recordings were performed in all NF1 patients with a diagnosis of epilepsy. Electrodes were placed in accordance with the International 10–20 system of placement as recommended by the International Federation of Clinical Neurophysiology (IFCN) [16]. The frequency filters used during recording were a low-pass filter 70 Hz and a high-pass filter 1 Hz. The standard duration of the EEG recording was 20 min awake and 30 min asleep [17]. Activation methods were used; intermittent photic stimulation (IPS) was applied in all patients, while in cooperative patients, it was during five minutes of hyperventilation [18]. Cognitive impairment was defined as an IQ lower than 70.

Pharmacoresistant epilepsy is defined by the ILAE as the lack of seizure freedom after trialing at least two appropriately chosen and adequately dosed ASMs.

The drugs used as a rescue medication for stopping the seizure were benzodiazepines as a first line drug (rectal diazepam (0.5 mg/kg), buccal midazolam (0.3 mg/kg), and parenteral (0.2 mg/kg) midazolam) and, as the second line drugs, parenteral phenobarbital (20 mg/kg) or levetiracetam (40–50 mg/kg). Depending on the clinical presentation, EEG finding, and response to initial ASMs, further epilepsy treatment was conducted. The patients were divided in two groups: epilepsy—NF1 patients (14) and non-epilepsy—NF1 patients (104). To evaluate the predictive factors (sex, intellectual impairment, UBOs, optic pathway glioma, hydrocephalus, vasculopathy, brain atrophy, head plexiform neurofibroma with/without intracranial propagation) for epilepsy in NF1 patients, univariate and multivariate logistic regression analyses were performed. Firth’s penalized likelihood correction was applied to prevent data separation. The model was fitted using the logistic package in R (version 1.26.1), and the predictive strength was expressed as odds ratios and *p* values. A multivariate logistic regression analysis was performed to obtain ORs and *p*-values for hydrocephalus and brain atrophy, adjusted for each other.

The study was conducted in accordance with the Declaration of Helsinki and approved by the Ethics Committee of Institute for Mother and Child Healthcare of Serbia (approval code 8/89, approved on 23 August 2025). Informed consent was obtained from all identifiable human participants.

## 3. Results

**Figure 1:**
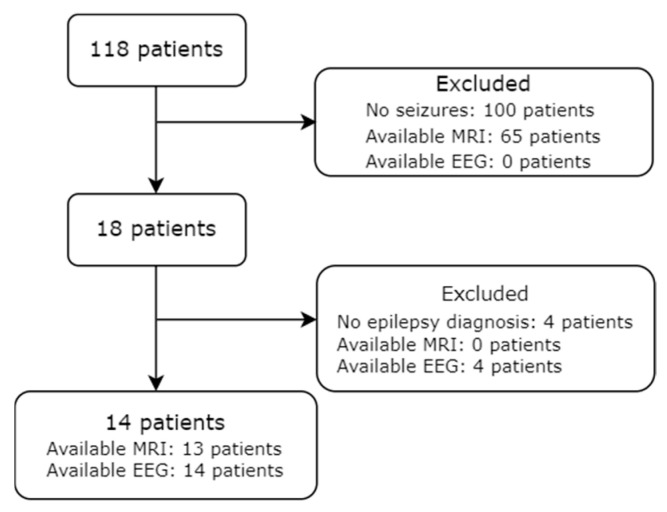
Flowchart illustrating the cohort of epilepsy-NF1 patients with data about EEG and MRI availability.

The study included 118 children with NF1. Seizures were experienced by 18 children (15.3%), while in 14 children (11.9%), a diagnosis of epilepsy was established (Figure 1).

The initial seizures had a focal onset in eight children, generalized onset in five children, while in one case, epileptic spasms were the first presentation of epilepsy. The mean age at onset of the first seizures was 59.3 ± 47.2 months (range 1–166), including one newborn with seizure onset on the fifth day of life. Ten children out of fourteen with an established diagnosis of epilepsy (71.4%) experienced convulsive SE as the initial epileptic event. Focal SE was observed in seven patients, with generalized tonic–clonic seizures in three patients. The mean duration of SE was 23 ± 12 (range 5–60) minutes.

The first seizure was stopped by the rectal administration of diazepam in seven cases, by intravenous or buccal midazolam in two, by phenobarbital in two patients, and in three cases, the seizures stopped spontaneously. In 14 children, in whom the diagnosis of epilepsy was established, ASM was recommended. The mean number of used ASMs was two with a range of 1–10 ASMs. Valproate was used in seven patients, carbamazepine in five, levetiracetam in four patients, clobazam in three, while in a single patient, phenobarbital, clonazepam, ethosuximide, primidone, phenytoin, and pregabalin were used. Five cases suffered from pharmacoresistant epilepsy, with a tendency to improve during the treatment. At the end of follow-up, with a mean duration of 62 months (range 4–150), good seizure control was achieved in all patients except one girl with initial epileptic spasms followed by focal epilepsy, resistant to ASMs. Although the course of epilepsy is favorable, seven (50.0%) out of fourteen patients required more than one ASM to achieve seizure control (Table 1).

In all cases with epilepsy, at least one video EEG recording was performed. The initial interictal EEG is presented in Table 1. The background activity was normal in ten patients and abnormal in four patients. The initial EEG showed diffuse epileptic discharges in 50% (7/14) and focal epileptic discharges in three cases; and four patients had normal initial interictal EEG findings.

Brain MRI was performed in 11 of 14 NF1 patients with epilepsy, and in 63 of 104 patients without epilepsy. The findings of the brain MRI showed abnormalities in a high number of patients in both groups: in nine of eleven (81.8%) patients with epilepsy and 52 of 63 (90.5%) without epilepsy. Unidentified bright objects dominantly localized in the basal ganglia and cerebellum were more frequently described in non-epilepsy group (52 of 63) than in patients with epilepsy (3/11), and the difference is statistically significant (OR = 0.22; *p*-value = 0.015). Optic glioma was registered in a similar percentage in both groups: 16.6% in NF1 group with seizures and 19.0% in NF1 patients without seizures, and the difference was not statistically significant (*p* = 0.73). Head plexiform neurofibromas (PNs) with/without intracranial propagation were present only in the NF1 group without epilepsy; so, head PNs are not a risk factor for seizures (*p* = 0.47) (Table 2).

**Figure 2:**
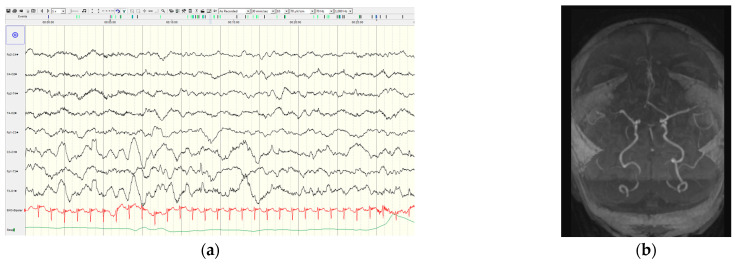
Electroencephalogram and magnetic resonance angiography in an NF1 newborn with neonatal-onset seizures: (**a**) interictal electroencephalogram at the age of 4 days showed asymmetric background activity with slowing and decreased amplitude above the right hemisphere; (**b**) magnetic resonance angiography at the age of 19 days showed a diminished signal in the A1 segment of the right anterior cerebral artery.

The first patient with a described ischemic stroke was a newborn with NF1 and epilepsy, who experienced their first seizure on the fifth day of life, and the seizures were repeated during the first two weeks. Since the seizures were resistant to phenobarbital, levetiracetam and clobazam were introduced leading to good seizure control. Unilateral slowing of background activity and focal discharges above the right hemisphere were recorded by video EEG. The brain MR showed ischemic lesion in the right frontal region of unknown etiology (Figure 2). A metabolic and thrombophilia workup were unremarkable. Whole exome sequencing revealed a pathogenic variant (c.6854dupA (p.Tyr228Ter)) in the *NF1* gene.

The second patient with a stroke was a 12-year-old boy with a first stroke at the age of two years, when he experienced first focal onset status epilepticus. At the age of six years, the boy experienced a second stroke. The MR angiography demonstrated marked stenosis of both supra-clinoid portions of the internal carotid arteries, with absent flow signal in the middle cerebral arteries and anterior cerebral arteries and extensive moyamoya collaterals. Brain perfusion MR studies revealed areas of reduced perfusion in both the right and left hemispheres. Despite extensive brain abnormalities, good seizure control was achieved by levetiracetam.

We found statistically significant predictive values of hydrocephalus (*p* = 0.001), brain atrophy (*p* = 0.002), and vasculopathy (*p* = 0.015) for epilepsy in children with NF1 (Table 3). Intellectual impairment was more frequent in the patients with epilepsy, showing a trend toward to significance (*p* = 0.065). In our cohort, the sex, presence of optic glioma, head plexiform neurofibroma with/without intracranial propagation, and UBOs were not significantly related with epilepsy (Table 3). The odds and *p* values obtained for hydrocephalus and brain atrophy, adjusted for each other, were both (60.2 and 0.001), respectively (Table 4).

## 4. Discussion

According to the literature data, the prevalence of epilepsy in NF1 patients is very variable, depending on the age, with a higher frequency in the pediatric population [7,8,9]. Santoro et al. reported a lower prevalence of epilepsy (4.3%) in a large cohort of 437 children with NF1 [19]; however, this is still ten times higher than in the general population (0.45%). In our cohort, 15.2% of children with NF1 experienced at least one seizure, and 11.9% were suffering from epilepsy. A higher rate of epilepsy in our cohort might be explained by the fact that all the evaluated patients were referred to a neurologist, due to neurological problems or cutaneous changes.

Although the course of epilepsy was favorable, seven (50.0%) out of fourteen patients required more than one ASM to achieve seizure control. In one patient with infantile spasms as the initial presentation of epilepsy, seizure control was not achieved even with ten different ASMs during the course of epilepsy. These results are similar to a study conducted by Ostendorf et al., which reported that about 90% of patients were treated with at least one anti-seizure medication (ASM), while 36% required treatment with two or more ASMs [10]. The results from a cohort of 33 NF1 patients with epilepsy showed that the majority of patients were seizure-free with monotherapy, while 11.5% needed more than one drug, including epilepsy surgery performed in three of thirty-three cases [6].

An association between NF1 and West’s syndrome was described in the literature with a favorable outcome [6,20] or was followed by resistant seizures, which could not be controlled by polytherapy [10,21,22]. Our patient with West’s syndrome was suffering from very resistant seizures requiring ten different ASMs, despite which, seizure control still was not achieved.

Previous studies documented that most seizures in NF1 patients are classified as focal onset seizures [11,21], similar to our cohort.

The understanding of epileptogenesis in neurofibromatosis is still insufficient. Commonly, epilepsy is associated with intracranial lesions, but 15–50% are non-lesional or do not correlate with brain abnormalities [10,11]. The literature data suggests that, in about 70% of cases, seizures arose in the context of neuroradiological findings, mainly cerebral vasculopathies and hydrocephalus [6]. The authors found that the prevalence of hydrocephalus (14.7%), cerebral vasculopathies (14.7%), and non-OPG brain neoplasm (29.4%) in patients with epilepsy were significantly higher than in patients without epilepsy [6]. In our cohort, in NF1 patients with epilepsy, hydrocephalus was found in three cases and vasculopathies in two (including one with moyamoya disease), while in the nonepileptic group, none of the patients had those MRI findings. We found that the predictive factors for epilepsy in children with NF1 were the presence of hydrocephalus, brain atrophy, and vasculopathy. Intellectual impairment was more frequent in patients with epilepsy, showing a trend toward to significance. The sex, presence of optic glioma, head plexiform neurofibroma with/without intracranial propagation, and UBOs were not significantly related with epilepsy. Our findings are similar to some previous studies, which showed no correlation between seizures and UBOs or optic gliomas in NF patients [10,21,23].

Neonatal onset seizure in NF1 patients has not been described in the literature until now, including the study of Santoro et al., which evaluated more than four hundred children with NF1 (19). Our case includes the first reported newborn with neonatal seizures followed by repeated focal seizure who required three different ASMs to achieve seizure control. The etiology of stroke in this case is still unclear, and all common risk factors for stroke at this age were excluded. Generally, the age of seizure onset in our cohort is mildly younger (five years) than in the literature [10,24,25].

Santoro et al. suggested that in the cases with NF1 and epilepsy, it is necessary to exclude brain tumors, as well as hydrocephalus and vasculopathy as the etiology of seizures in NF1 children (19).

The results of our study might contribute to a better understanding of epilepsy in NF1 cases, and some unpublished data are pointed to in our work, such as the high frequency of status epilepticus, neonatal seizures as initial epilepsy presentation in NF1, report of electro-clinical features, and risk factors for epilepsy in children with NF1.

The first limitation of our study is the insufficient number of patients with available brain MRI findings, especially in cases with epilepsy and NF1. The second limitation is the insufficient number of genetic analyses, which enable an analysis of the genotype–phenotype correlation in terms of epilepsy, although some reports suggest no correlation [6]. In addition, a limitation of the study is the lack of data in terms of the correlation between behavior problems and epilepsy.

## 5. Conclusions

In our cohort of NF patients, epilepsy was at least ten times more frequent than in the general population. The recommendation for rescue medication and parental education is very important since a high percentage of NF1 patients with seizures were suffering from status epilepticus and, therefore, required prompt medical attention. The outcome of epilepsy was favorable in all cases, except one girl with initial epileptic spasms, although, nearly half of the patients required more than one ASM to achieve seizure-free status. It seems that presence of seizures does not directly contribute to cognitive impairment, since in almost all cases, the epilepsy was well controlled. NF1 children with hydrocephalus, brain atrophy, and vasculopathy, described on brain MRI, are at a higher risk for epilepsy. Further investigation is needed to better understand epileptogenesis and the factors that influence the clinical course of epilepsy in NF1.

## Figures and Tables

**Table 1:** Demographic, clinical, and electroencephalographic characteristics in children with neurofibromatosis type 1 and epilepsy.

N	Sex	Age at First Seizure (Months)	Seizure Classification	Status Epilepticus (Y/N)	No. of ASM	Initial EEG Interictal Findings (BA, ED)
1	F	1	Focal	Y	3	Asymmetric theta BA, with slow delta and sharp-slow waves above right hemisphere
2	F	50	Focal	Y	1	Normal BA and focal ED in the centro-temporal right region spreading to the left side
3	M	156	GTC	N	1	Normal BA and diffuse spike-waves
4	F	5	ES	Y	10	Irregular BA, diffuse and multifocal spikes-waves, and polyspikes-waves
5	M	161	GTC	N	4	Normal BA, diffuse spike-waves, and polyspikes-waves
6	F	50	Atonic	N	4	Normal BA and no ED
7	F	24	Focal	Y	1	Normal BA and bilateral paroxysms of slow sharps and waves
8	M	11	Atonic	Y	2	Slow BA and diffuse ED
9	F	72	Focal	Y	2	Normal BA and diffuse sharp waves
10	M	41	Focal	Y	1	Normal BA and right parieto-temporal sharp waves
11	M	41	Focal	Y	1	Normal BA and no ED
12	F	50	GTC	N	1	Normal BA and no ED
13	F	120	Focal	Y	1	Normal BA and no ED
14	M	24	Focal	Y	3	Slow BA and diffuse ED

Abbreviations: GTC: generalized tonic–clonic, ES: epileptic spasms, ASM: antiseizure medication; BA: background activity; EEG: electroencephalogram; ED: epileptic discharges.

**Table 2:** Neuroimaging and cognitive characteristics in children with neurofibromatosis type 1 and epilepsy.

N	Sex	Cognitive Impairment	Brain MRI
1	F	No	Ischemic stroke in right frontal region
2	F	No	UBOs, left optic glioma
3	M	Yes	UBOs
4	F	Yes	Supratentorial atrophy, thinned corpus callosum, UBOs
5	M	Yes	White matter atrophy
6	F	Yes	Normal MRI
7	F	No	Chiari malformation type 1
8	M	No	Hydrocephalus, bilateral optic glioma
9	F	No	Cerebellar arachnoid cyst
10	M	Yes	Optic glioma
11	M	No	UBOs, supratentorial atrophy, Arnold Chiari malformation type 1
12	F	No	N/A
13	F	No	UBOs
14	M	Yes	MR angiography—stenosis of both ICA, absent flow signal in ACM and ACA, extensive moyamoya collaterals

Abbreviations: ICA—internal carotid artery, ACA—anterior cerebral artery; UBO—unidentified bright objects.

**Table 3:** Univariate analyses of the predictive factors for epilepsy in NF patients.

Factor	Coefficient (β)	Odds Ratio (OR)	*p* Value
Sex	0.1173	1.12	0.833
Hydrocephalus	3.7766	43.67	0.001
Head PN	−0.9378	0.39	0.471
Optic Glioma	−0.2296	0.80	0.730
UBO	−1.5012	0.22	0.015
Intel. Impairment	1.0885	2.97	0.065
Ischemic Stroke	3.3491	28.46	0.010
Brain Atrophy	3.7612	43.05	0.002

Abbreviations: UBO—undefined bright objects; PN—plexiform neurofibroma.

**Table 4:** Multivariate analysis of the predictive factors for epilepsy in NF1 patients.

Factor	Coefficient (β)	Std. Error	*p* Value	Odds Ratio (OR)	95% CI for OR
Hydrocephalus	4.0976	1.5603	<0.001	60.2	5 to 8413
Brain Atrophy	4.0976	1.5603	<0.001	60.2	5 to 8413

## Data Availability

The original contributions presented in this study are included in the article. Further inquiries can be directed to the corresponding author.
